# Residual efficacy of selected larvicides against *Culex pipiens pipiens* (Diptera: Culicidae) under laboratory and semi-field conditions

**DOI:** 10.1007/s11356-022-24654-6

**Published:** 2023-01-10

**Authors:** Maria K. Sakka, Charalampos S. Ioannou, Nikos T. Papadopoulos, Christos G. Athanassiou

**Affiliations:** Laboratory of Entomology and Agricultural Zoology, Department of Agriculture, Crop Production and Rural Environment, University of Thessaly, Phytokou Str., 38446 Nea Ionia, Magnesia Greece

**Keywords:** *Bti*, Control, Diflubenzuron, Larvicides, Mosquitoes, Polydimethylsiloxane, Temephos, S-methoprene, Spinosad

## Abstract

Mosquitoes are a threat worldwide since they are vectors of important pathogens and parasites such as malaria, dengue, yellow fever, and West Nile. The residual toxicity of several commercial mosquito larvicides was evaluated for the control of *Culex pipiens pipiens* under controlled laboratory and semi-field conditions during late spring and summer of 2013. The evaluation included six different active ingredient formulations, i.e., diflubenzuron Du-Dim), *Bacillus thuringiensis* var. *israelensis* (*Bti*) (Vectobac), spinosad (Mozkill), S-methoprene (Biopren), temephos (Abate), and polydimethylsiloxane (PDMS) (Aquatain), that are currently registered of and had been registered in the past for mosquito control. Under controlled laboratory conditions, the residual activity ranged from 1 week (S-methoprene) up to 2 months (spinosad, PDMS). Exposure of larvicides under semi-field conditions resulted in noticeable differences regarding their efficacy as compared to the laboratory bioassays. Exposure of S-methoprene, *Bti*, and spinosad, for up to 3 days, resulted in similar adult emergence to the controls. On the other hand, the residual efficacy of diflubenzuron, temephos, and PDMS ranged from 14 to 28 days, depending on the season of exposure. Longevity and fecundity of adults that had emerged from surviving larvae, in most of the cases tested, did not differ significantly from that of the controls. The results of the present study demonstrate the necessity of both field and laboratory studies to draw safe conclusions regarding the efficacy of larvicides against mosquitoes and the selection of the proper formulation for each application scenario. In addition, defining the seasonal variation in the residual toxicity of the tested formulations could be useful for improving mosquitos’ management programs.

## Introduction

*Culex pipiens* L. (Diptera: Culicidae) is a competent vector of several human and animal diseases, including filariasis and arboviruses, such as West Nile virus (WNV), Sindbis virus, Rift Valley fever, and Japanese encephalitis virus (Farajollahi et al. 2011). Although in recent years several non-chemical methods for mosquitoes’ control have been developed and successfully tested, insecticide applications still remain the principal tool for tackling *Cx. pipiens* populations and associated diseases (WHO 2013). In this context, extensive application of larvicides is considered the most important tool for the prevention of mosquito borne diseases, since it targets immature stages and thus prevent females’ emergence which are responsible for the pathogens’ transmission. Larvicides are applied directly on the water surface to control developing larvae, and therefore, their efficacy is highly depended on their residual activity. Nevertheless, an ideal larvicide should also combine high efficacy against mosquitoes with low toxicity against non-target organisms, including humans. In this regard, only few active ingredients are still in use, especially in developed countries under the current biocide legislations and regulations.

Several active ingredients have been used as larvicides for the suppression of mosquito populations. One of the most used compounds is the organophosphate temephos, which acts through the acetylcholine receptors in the insects’ nervous system. Despite its high efficacy and fast mode of action, the use of temephos was phased out from the European Union (EU) more than 10 years ago (Baldacchino et al. 2015). However, the persistence of WNV human infections in several EU Member States every year (ECDC) has already opened up the discussion for reconsidering “traditional” larvicides as an emergency tool for the prevention of possible outbreaks of human diseases (Afrane et al. 2016; Derua et al. 2018) In the recent years, insect growth regulators (IGRs), such as the chitin synthesis inhibitor diflubenzuron and the mimic hormone analogue S-methoprene, became probably the most commonly used mosquito larvicides globally, as they combine high persistence with low mammalian toxicity (Russell and Kay 2008). *Bacillus thuringiensis* subsp. *israelensis* (*Bti.*) (Bacillales: Bacillaceae), which acts to the mosquito larvae through digestion are considered as environmentally friendly larvicides. Spinosad that is based on metabolites of the actinomycete *Saccharopolyspora spinosa* has been proved very effective against different species of mosquitoes as well (Hertlein et al. 2011). Recently, several inert materials such as the polydimethylsiloxane (PDMS) have been also assessed successfully as larvicides, due to their incontestable advantages, which combine low levels of interaction with the environment, mechanical (non-neurotoxic) mode of action, and considerable residual activity (Bukhari and Knols 2009; Mbare et al. 2014; Rumbos and Athanassiou 2020; Nikolaidou et al. 2021). Due to its physical mode of action PDMS (a monomolecular film, which act on mosquitoes by closing off their respiratory structures and finally leading to suffocation) and its friendly environmental profile, many countries have exempted it from formal registration as a biocide (Bukhari and Knols 2009; Nikolaidou et al. 2021). With the exception of temephos and spinosad, which are not registered for mosquito control in EU anymore, diflubenzuron, S-methoprene, *Bti*, and PDMS are extensively used in many EU countries (Grigoraki et al. 2017; Bruehl et al. 2020; Nikolaidou et al. 2021).

The success of mosquito control programs relies heavily on the effectiveness of the insecticides after application, i.e., their residual toxicity. In general, larvicides efficacy can be affected by a series of environmental factors such as temperature, sunlight, water organic matter, aquatic vegetation, and rain (Brar et al. 2006; Devillers 2020). At the same time, the selected active ingredients should be in compliance with certain legislative constraints, such as areas that fall in the category of protected aquatic ecosystems, where most of the conventional compounds cannot be applied. Considering all these factors as well as the limited number of available active ingredients, there is a need to compare their efficacy under “common garden” conditions.

In the vast majority of the studies, the insecticidal effect of mosquito larvicides is examined under laboratory conditions, while there is still inadequate information regarding their field performance based on experimentally sound studies. Moreover, even when different larvicides are compared in the laboratory, these data are not expanded to further testing under “controlled” field conditions, i.e., the so called “semi-field” tests, despite the fact that there is strong evidence that different insecticides behave in a dissimilar way when they are applied in the field (Msangi et al. 2011; Rumbos and Athanassiou 2020). In this context, the results for laboratory experiments should be coupled with field and semi-field tests, as many larvicides are heavily influenced by biotic and abiotic conditions that are not present in the laboratory. Moreover, these tests should be performed at the area or interest, i.e. the area that is to be treated, taking into account the individual conditions and the microenvironment, such as physicochemical properties of water and weather conditions. The aim of the current study was to carry out simultaneous laboratory and semi-field trials for a wide range of larvicides with dissimilar modes of action, in order to quantify their insecticidal and residual effect against *Cx. pipiens*.

## Materials and methods

### Mosquito rearing and control agents

We used a strain of *Cx. pipiens* biotype *pipiens* that had been collected for the area of Volos Greece and had reared for several generations under laboratory conditions. The mosquitoes were sampled from a collection of 300 egg rafts from basins with water which were settled in the surroundings of the Department of Agriculture, Crop Production and Rural Environment, at the University of Thessaly from 15 to 30 June of 2012. The experiments were conducted in the Laboratory of Entomology and Agricultural Zoology, Department of Agriculture, Crop Production and Rural Environment, University of Thessaly (Nea Ionia, Magnesia, Greece), in incubators set at 24.05 ± 0.5 °C, 75–80% relative humidity, and under a photoperiod of 14:10 (L:D). The larvicides used in this study and the respective concentrations are given in Table 1.

**Table 1:** Larvicides formulations tested in this study, their active ingredients, and the respective providers

Formulation	Active ingredient	Provider
Du-Dim 15 SC	Diflubenzuron	Arysta, Greece
Oprah 15 SC	Diflubenzuron	Farma-Chem, Greece
Mozkill 120 SC	Spinosad	Elanco, Greece
Vectobac 12 SC	*Bacillus thuringiensis*	BASF, Greece
Biopren BM 20	S-Methoprene	Farma-Chem, Greece
Abate	Temephos	BASF, Greece
Aquatain AMF	Polydimethylsiloxane	Dafni SA, Greece

We used *F*_3-10_ individuals of the laboratory colony. *Culex pipiens* larvae were reared in pans 50 × 30 × 14 cm containing about 8 l of water. In each pan, we placed about 250–300 egg rafts. Food was given in the form of a powdered cat meal (Friskies adult, Purina®) on a daily basis. Pupae where then collected and introduced into special plastic containers and placed in the adult rearing cages (30 × 30 × 30 mm). The number of pupae per cage was about 700–1000. The adults were provided with cotton balls soaked in a 10% sugar solution. Five to seven days after emergence, chicken blood was offered as a source for blood meal to adult females, and 1 week later, the eggs were collected in a plastic container with 750 ml of water and 0.3 gr of food. Twenty-four hours later, the egg rafts that had been deposited were transported with the help of filter paper to the pans of development.

### Larvicidal bioassays

#### Laboratory efficacy assessment

The bioassay method followed was based on the standard test for determining the susceptibility of mosquito larvae to insecticides (WHO 1981). Specifically, plastic containers were used with 150 ml of water and 15 mg of food daily. The rate was calculated according to a surface of 46.54 cm^2^ (see Table 2). Firstly, the containers were filled with 100 ml of water and 15 mg of food and in each of the container we applied the recommended dose of each larvicide corresponding to the above surface. The containers were sealed with a membrane which had 5 holes of 0.5 mm each to minimize evaporation and kept at the same conditions until the beginning of bioassays. Each series of bioassays consisted of 20 third instar larvae with 50 ml of water in each series of containers, performed at weekly intervals. For each treatment and the control, there were 6 replicates. Efficacy was recorded according to the number of adults emerged.

**Table 2:** Doses of larvicide formulations applied during the evaluation

Formulation	Label range	Doses	Dose corresponding to the surface of 46.54 cm ^2^
Du-Dim 15 SC	3.3–6.6 ml/100 m^2^	4.95 ml/100 m^2^	0.23 µl
Oprah 15 SC	3.3–6.6 ml/100 m^2^	4.95 ml/100 m^2^	0.23 µl
Mozkill 120 SC	45 ml/1000 m^2^	45 ml/1000 m^2^	0.21 µl
Vectobac 12 SC	80–200 ml/1000 m^2^	140 ml/1000 m^2^	0.65 µl
Biopren BM 20	60 ml/10000 m^2^	60 ml/10,000 m^2^	0.028 µl
Abate	15–20 ml/1000 m^2^	17.5 ml/1000 m^2^	0.08 µl
Aquatain AMF	1000 ml/100 m^2^	1000 ml/100 m^2^	4.70 µl

#### Efficacy assessment in semi-field conditions

The methodology (rearing and bioassays) followed was the same as in the previous test with the only difference that the containers were sealed with plastic caps to protect it from rain and wind. Containers were sealed and placed outside, at the beginning of May, June, and July 2013 (separate series of containers for each month). Efficacy was recorded at weekly intervals, as in the previous tests. In these tests the preparation of Oprah 15 SC was not used since the laboratory tests indicated similar efficacy levels with Du-Dim 15 SC (same active ingredient).

During these bioassays, in cases where mortality was < 60%, the adults (both males and females) emerged were transferred back to walk-in chambers and placed in plexiglass boxes (20 × 20 × 20 cm) and provided with 10% sugar solution to assess survival over a period of 28 days post-emergence. Furthermore, blood-feeding was carried out on the 20th day (after emergence) for 30 min. Seven days later, the containers were filled with water, and a small amount of food was provided to stimulate oviposition. On the 28th day after the end of the assessed adult survival, the containers were removed, and the number of egg rafts that had been oviposited by females, both in the controls and the treatments, were recorded. Three days later, the egg rafts hatched were recorded. The average temperature of each day for May, June, and July was 23.06, 24.50, and 27.14 °C, respectively.

### Statistical analysis

For the laboratory trials, *t*-test was performed to compare mortality in treated and control pots. *t*-test was performed for the comparisons in pairs (treated-control) when required. One-way ANOVA was considered to analyze the semi-field trials mortality data followed by the post hoc test Tukey’s HSD. Before the analysis, normality and homogeneity were checked, when necessary, and data were properly transformed. When all transformation failed, data were submitted to the nonparametric Kruskal–Wallis *H*-test, and the means were separated by the Mann–Whitney *U*-test. Chi-squared test was used for survival rates of treated and control adults, rates of females who laid eggs, and rates of hatching followed by individual analyzes for comparisons by pairs. For the larvicides that showed remarkable stability (Du-Dim, Abate, and Aquatain), mortality data were subjected to repeated measures to determine the effect of exposure, the type of larvicide, and their interaction with their effectiveness for each month. All analyses were performed with the statistical package SPSS version 16 (SPSS Inc., Chicago, IL), and significance level was set at *α* = 0.05.

## Results

### Laboratory bioassays

**Fig. 1:**
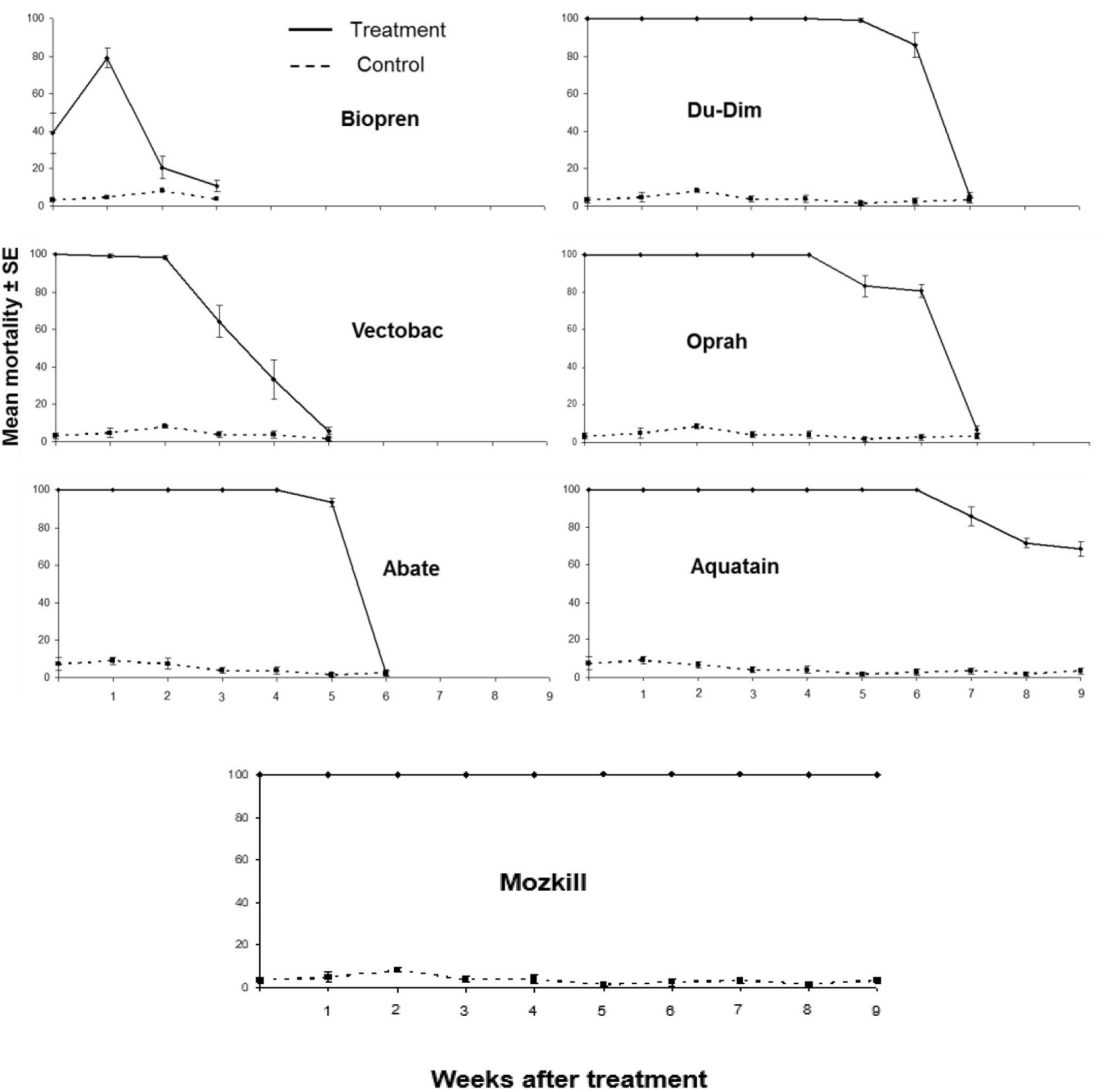
Efficacy of formulations with respect to time following their application under laboratory conditions

The efficacy of the formulations applied under laboratory conditions showed significant variations, ranging from a week for Biopren to about 2 months for Mozkill and Aquatain (Fig. 1). Interestingly, for Biopren, mortality was increased 1 week after the application. This is probably due to the absorption of the formulation from food during this period and the efficient intake of it by larvae. However, 3 weeks after the application, its efficacy decreased dramatically, and the mortality of larvae did not differ significantly in comparison with the control (*t* = 1.98, df = 10, *P* = 0.076). Vectobac and Abate mortality levels were decreased 5 and 6 weeks after the application, respectively (*t* = 1.84, df = 7.56, *P* = 0.096 and *t* = 0, df = 10, *P* = 1, respectively). As expected, Du-Dim and Oprah showed a similar efficacy level, with high mortality rates for 6 weeks after the application (*t* = 1.10, df = 10, *P* = 0.296 and *t* = 1.24, df = 10, *P* = 0.243, respectively). Similarly, Aquatain and Mozkill were also highly effective for up to 9 weeks after the application with the average mortality rate being around 70 and 100%, respectively (Fig. 1).

### Field bioassays

#### May field test

**Fig. 2:**
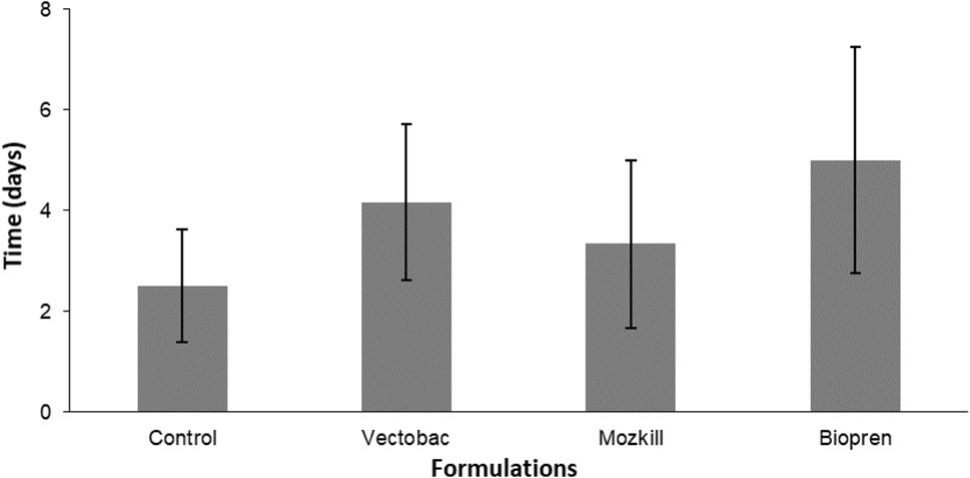
Efficacy of Vectobac, Mozkill, and Biopren during the first week of exposure in the May field test

**Fig. 3:**
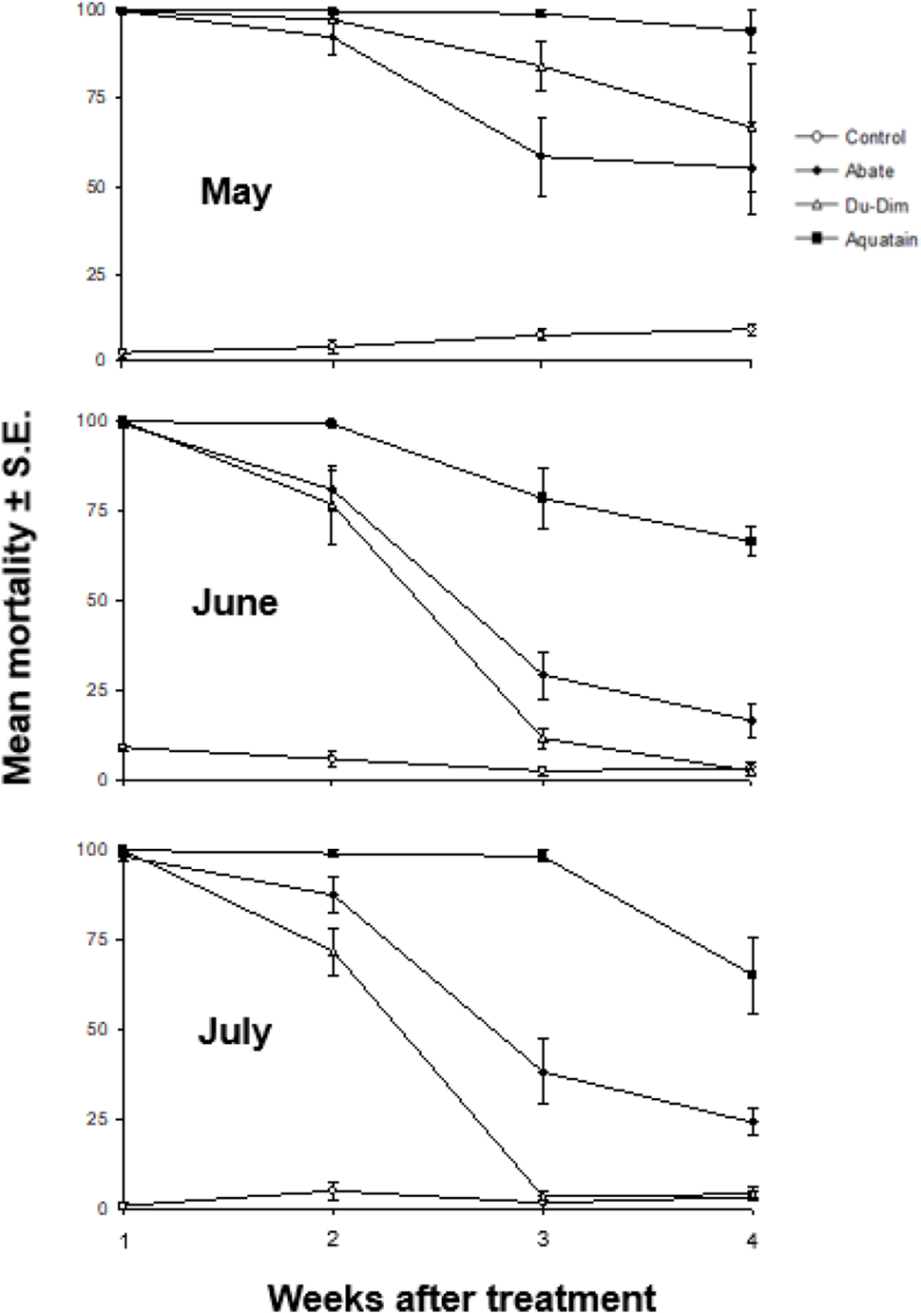
Efficacy of the formulations Du-Dim, Abate, and Aquatain in field tests for the months May, June, and July

Exposure of formulations during the first week of May indicated that the mortalities caused by Biopren, Vectobac, and Mozkill were extremely low, with no significant differences among treatments including control (*F* = 0.407, df = 3, *P* = 0.750) (Fig. 2). Furthermore, no significant differences were noted in the case of the biological parameters of adults in comparison with the control (Table 3) . In contrast, Du-Dim, Abate, and Aquatain showed high efficacy with mortality exceeding 50% even 4 weeks after the application (Fig. 3) . In this case, there was a significant effect of exposure time (*F* = 7.038, df = 17.29, *P* = 0.014), and the type of formulation (*F* = 21.439, df = 2, *P* < 0.001) but not of their interaction (*F* = 1.623, df = 2.30, *P* = 0.225). Comparisons on the efficacy of these formulations indicated significant differences between them during the third week of the bioassays (*x*^2^ = 10.30, df = 2, *P* = 0.006). Moreover, Aquatain differed significantly from Du-Dim and Abate (*U*_6_,_6_ = 7, *P* = 0.050 and *U*_6.6_ = 0, *P* = 0.003, respectively), while no significant difference was observed between Du-Dim and Abate (*U*_6.6_ = 7.5, *P* = 0.090). Comparisons for the fourth week of May also showed significant differences between formulations (*x*^2^ = 6.45, df = 2, *P* = 0.040), where the efficacy of Aquatain varied significantly than that of Abate (*U*_6,6_ = 3, *P* = 0.012) but not than that of Du-Dim (*U*_6,6_ = 9, *P* = 0.107), while no significant differences were observed between Du-Dim and Abate (*U* = 12, *P* = 0.328).

**Table 3:** Biological parameters of adults in the May field test with Vectobac, Mozkill, and Biopren (in percentage and absolute numbers). Biological parameters of the adults emerged from larvae that were developed both in control and in solutions of the formulations Vectobac, Mozkill, and Biopren that remained in the field during the first week of May

	Percentage %			
	Survival^1^		Ovipositing females^2^	Egg raft hatch^3^
	♂♂	♀♀		
Vectobac	22.22 (63)	82.7 (52)	67.4 (43)	100 (29)
Mozkill	36.5 (52)	90.6 (64)	69 (58)	95 (40)
Biopren	43.5 (62)	90.4 (52)	70.21 (47)	96.97 (33)
Control	27 (63)	96.3 (54)	69.23 (52)	94.44 (36)
*Χ*^2^	7.78	5.54	0.08	1.73
df	3	3	3	3
*P*	0.051	0.136	0.994	0.631

^1^The proportion of adults who survived until the 28th day of life. In parentheses the initial number of both sexes emerged in each case is given

^2^This parameter refers to females who managed to survive until the 28th day of their life. In parentheses the number of females which laid eggs is given

^3^In parentheses the number of “projects” eggs deposited is given

**Fig. 4:**
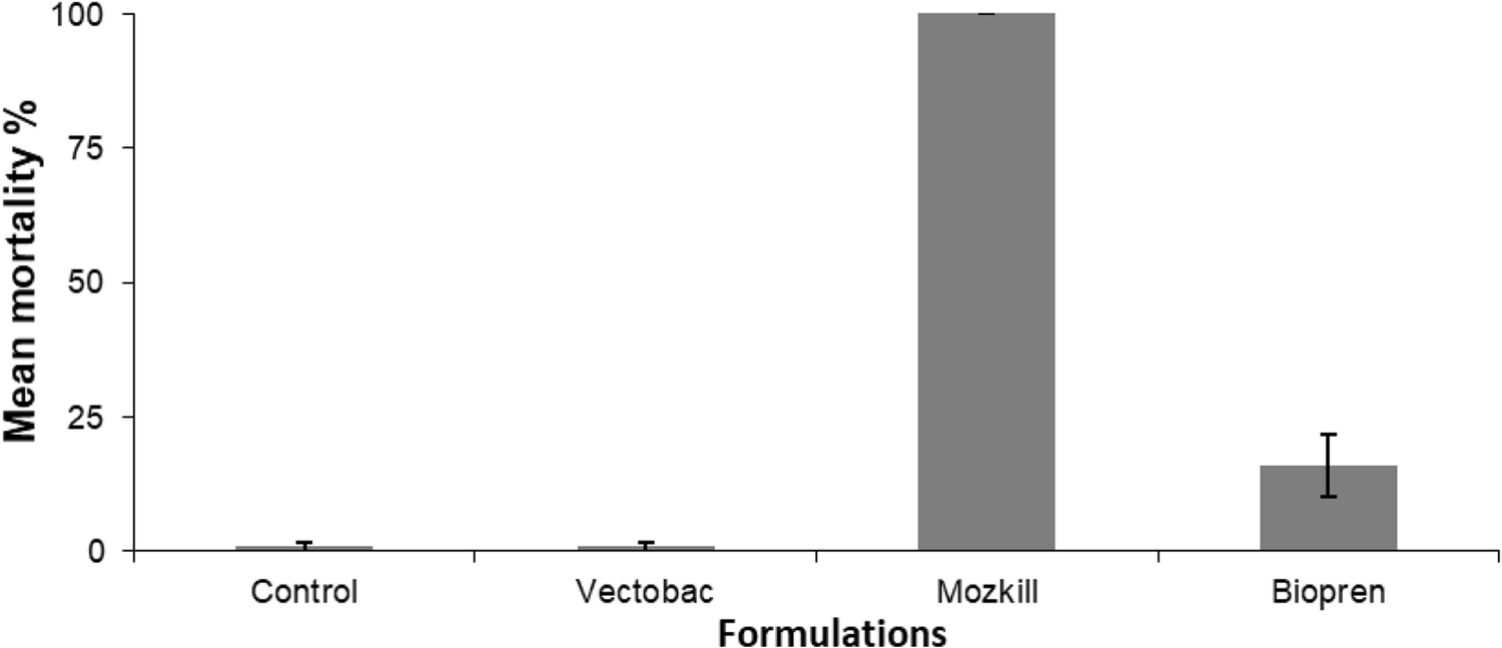
Efficacy of the formulations Vectobac, Mozkill, and Biopren in field tests for the first day of June (*Ρ* < 0.05, Tukey’s HSD test)

#### June field test

The efficacy of Vectobac, Mozkill, and Biopren on the first day of the June bioassays showed significant differences (*F* = 79.364, df = 3,20, *P* < 0.001) (Fig. 4). The biological parameters of adults emerged from the treatment of Vectobac, Biopren, and the control indicated significant differences in terms of survival of both sexes and the number of females which were allowed to lay eggs (Table 4). The average mortality after exposure to Mozkill on the first 3 days of June in the field was 22.5 ± 7.5 and did not differ significantly (*t* = 1.95, df = 5.52, *P* = 0.103) as compared to the control (7.5 ± 1.71). The biological parameters of adults that emerged in both cases showed significant differences only in male survival (Table 5) . The efficacy of Du-Dim and Abate showed a significant decline after 3 weeks, as opposed to Aquatain that showed a remarkable stability (Fig. 3). Differences in the biological parameters of adults among Du-Dim, Abate, and control after 3 weeks showed significant differences only in adult survival (Table 6). Repeated measurements in this case showed a significant effect on the efficacy of exposure time (*F* = 129.838, df = 1.83, *P* < 0.001), type of formulation (*F* = 69.487, df = 2,15, *P* < 0.001), and their interaction (*F* = 10.399, df = 3.65, *P* < 0.001). Comparisons among the three formulations showed significant differences in efficacy both at the third (*F* = 22.751, df = 2, *P* < 0.001) and in the fourth week (*F* = 56.186, df = 2, *P* < 0.001) (*P* < 0.05, Tukey HSD test). The biological parameters of adults emerged from the treatment of Du-Dim, Abate, and the control during the third week of June showed significant differences only in male survival (Table 6).

**Table 4:** Biological parameters of adults in the June field tests with Vectobac and Biopren (in percentage and absolute numbers). Biological parameters of the adults emerged from larvae that were developed both in control and in solutions of the formulations Vectobac and Biopren that remained in the field during the first day of June

	Percentage %			
	Survival ^1^		Ovipositing females^2^	Eggrafthatch^3^
	♂♂	♀♀		
Vectobac	47.62a (63)	87.5a (56)	34.78b (49)	81.25 (16)
Biopren	54.09a (61)	62.5b (40)	32b (25)	75 (8)
Control	29.85b (67)	78.85ab (52)	70.73a (41)	93.1 (29)
*Χ*^2^	8.30	8.48	15.58	2.41
df	2	2	2	2
*P*	0.016	0.014	< 0.001	0.300

^1^The proportion of adults who survived until the 28th day of life. In parentheses the initial number of both sexes emerged in each case is given

^2^This parameter refers to females who managed to survive until the 28th day of their life. In parentheses the number of females which laid eggs is given

^3^In parentheses the number of “projects” eggs deposited is given

**Table 5:** Biological parameters of adults in the June field test with Mozkill (in percentage and absolute numbers). Biological parameters of the adults emerged from larvae that were developed both in control and in solutions of the formulation Mozkill who remained in the field during the first 3 days of June

	Survival %			
	Survival^1^		Ovipositing female^2^	Egg rafts hatch^3^
	♂♂	♀♀		
Mozkill	34.21 (38)	80 (55)	75 (44)	96.97 (16)
Control	81.69 (71)	90 (40)	72.22 (36)	92.31 (29)
*Χ*^2^	24.57	1.74	0.08	0.66
Df	1	1	1	1
*P*	< 0.001	0.187	0.779	0.418

^1^The proportion of adults who survived until the 28th day of life. In parentheses the initial number of both sexes emerged in each case is given

^2^This parameter refers to females who managed to survive until the 28th day of their life. In parentheses the number of females which laid eggs is given

^3^In parentheses the number of “projects” eggs deposited is given

**Table 6:** Biological parameters of adults in the June field tests with Du-Dim and Abate (in percentage and absolute numbers). Biological parameters of the adults emerged from larvae that were developed both in control and in solutions of the formulations Du-Dim and Abate that remained in the field during the first 3 weeks of June

	Percentage %			
	Survival^1^		Ovipositing^2^	Egg raft hatch^3^
	♂♂	♀♀		
Du-Dim	43.75a (64)	88.09 (42)	51.35 (37)	94.74 (19)
Abate	25ab (40)	91.11 (45)	63.41 (41)	92.3 (26)
Control	20.29b (69)	87.5 (48)	71.43 (42)	100 (30)
*Χ*^2^	9.33	0.35	3.40	2.25
Df	2	2	2	2
*P*	0.009	0.841	0.182	0.324

^1^The proportion of adults who survived until the 28th day of life. In parentheses the initial number of both sexes emerged in each case is given

^2^This parameter refers to females who managed to survive until the 28th day of their life. In parentheses the number of females which laid eggs is given

^3^In parentheses the number of “projects” eggs deposited is given

#### July field tests

**Fig. 5:**
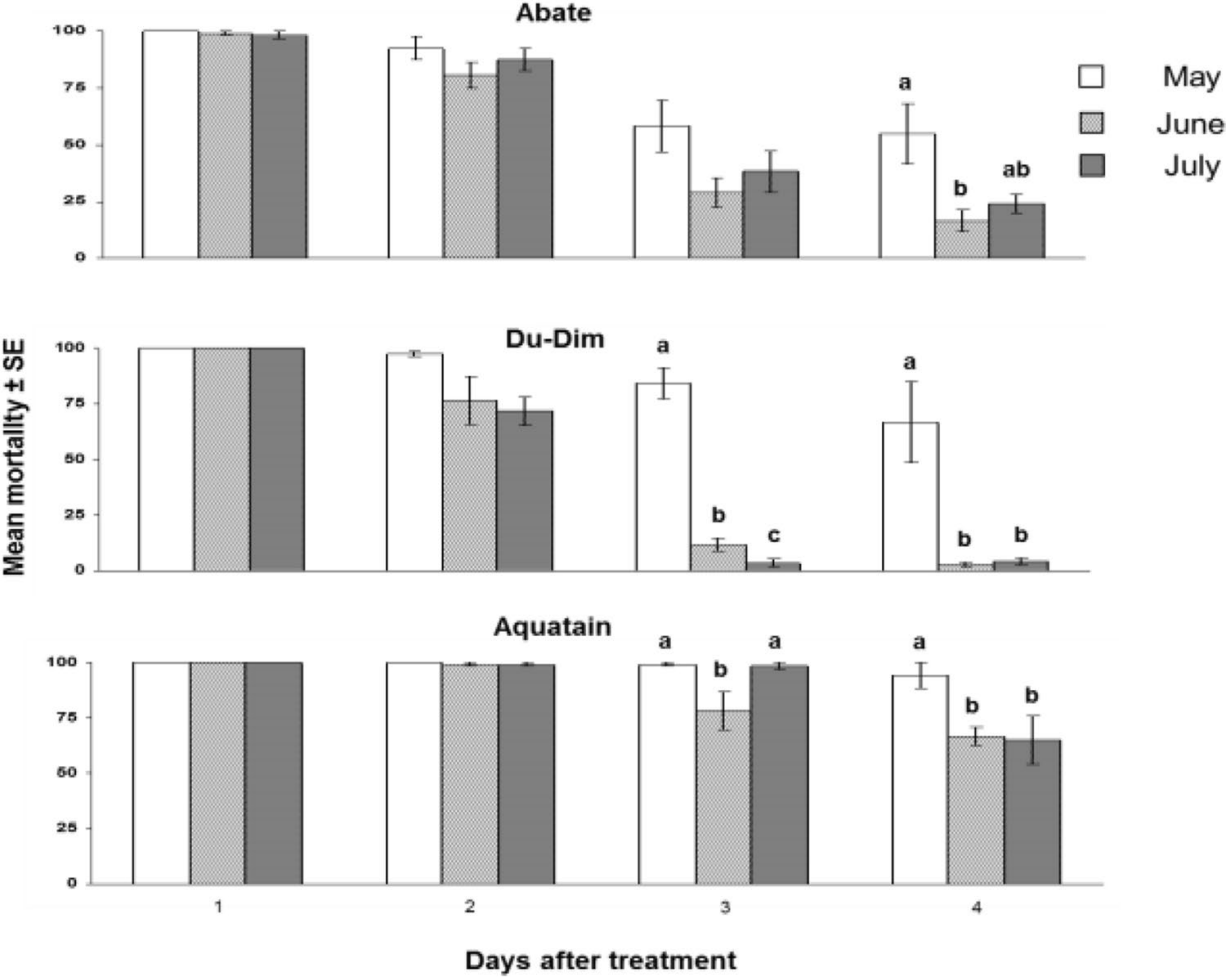
Comparison of the efficacy of formulations Abate, Du-Dim, and Aquatain for the months May, June, and July (*P* < 0.05, Tukey’s HSD test and Mann–Whitney *U* test for comparisons in Aquatain by the third week)

The exposure of Mozkill on the first day of July did not affect efficacy (average mortality 100%) as was the case for the month of June (see above). Moreover, an additional day resulted in an average drop of mortality rate to 42.50 ± 6.55 continuing nevertheless to differ significantly (*t* = 5.514, df = 10, *P* < 0.001) as compared to the controls (5.00 ± 1.83). The biological parameters of adults emerged in both cases, i.e., Mozkill and control, did not indicate significant differences (Table 7) . As in the case of May and June, Du-Dim and Abate showed a significant decline in efficacy at the third week of exposure and so on. In contrast, Aquatain had the best performance regarding its larvicidal effect (Fig. 3). Repeated measurements for the period of July for the above formulations showed a significant effect on efficacy of exposure time (*F* = 118.939, df = 3,45, *P* < 0.001), type of formulation (*F* = 110.337, df = 2, *P* < 0.001), and their interaction (*F* = 16.784, df = 6,45, *P* < 0.001). Comparisons between the three formulations showed significant differences in efficacy among them (mortality) during the second (*F* = 14.409, df = 2,15, *P* < 0.001), third (*F* = 93.351, df = 2.15, *P* < 0.001), and fourth (*F* = 19.531, df = 2,15, *P* < 0.001) week of exposure in the field. The biological parameters of adults emerged from the treatment of Du-Dim, Abate, and the control during the third week of July showed significant variations in the survival of both sexes and larval emergence (Table 8). Comparisons on the efficacy of these three formulations between the months of May, June, and July revealed significant differences in the last 2 weeks of exposure in the field (Fig. 5).

**Table 7:** Biological parameters of adults in the July field test with Mozkill (in percentage and absolute numbers). Biological parameters of the adults emerged from larvae that were developed both in control and in solution of the formulation Mozkill which remained in the field during the first 2 days of July

	Percentage %			
	Survival^1^		Ovipositing^2^	Egg raft hatch^3^
	♂♂	♀♀		
Mozkill	60.53 (38)	87.1 (31)	59.26 (27)	87.5 (16)
Control	47.14 (70)	90.9 (44)	70 (40)	92.86 (28)
*Χ*^2^	1.77	0.28	0.83	0.35
Df	1	1	1	1
*P*	0.184	0.598	0.364	0.552

^1^The proportion of adults who survived until the 28th day of life. In parentheses the initial number of both sexes emerged in each case is given

^2^This parameter refers to females who managed to survive until the 28th day of their life. In parentheses the number of females which laid eggs is given

^3^In parentheses the number of “projects” eggs deposited is given

**Table 8:** Biological parameters of adults in the July field tests with Du-Dim and Abate (in percentage and absolute numbers). Biological parameters of the adults emerged from larvae that were developed both in control and in solutions of the formulations Du-Dim and Abate which remained in the field during the first 3 days of July

	Percentage %			
	Survival^1^		Ovipositing^2^	Eggrafthatch^3^
	♂♂	♀♀		
Du-Dim	41.79b (67)	71.43b (49)	48.57 (35)	94.12ab (17)
Abate	87.04a (54)	80ab (20)	56.25 (16)	77.78b (9)
Control	25.35c (71)	93.62a (47)	59.01 (44)	100a (26)
*Χ*^2^	48.55	8.02	0.89	6.07
	2	2	2	2
*P*	< 0.001	0.018	0.641	0.048

^1^The proportion of adults who survived until the 28th day of life. In parentheses the initial number of both sexes emerged in each case is given

^2^This parameter refers to females who managed to survive until the 28th day of their life. In parentheses the number of females which laid eggs is given

^3^In parentheses the number of “projects” eggs deposited is given

## Discussion

The results of the present work clearly indicate the different performance of some of the insecticides tested between laboratory and semi-field tests. Hence, spinosad (Mozkill), which provided a very high level of efficacy during the laboratory tests, lost its larvicidal effect even from the first week of the field tests. It is generally recognized that spinosad can be dissipated rapidly after exposure to light (UV), which is an issue of concern in different application scenarios (Hertlein et al. 2011; Adak and Mukherjee 2016). In this context, we estimate that this remarkable loss in efficacy is due to the exposure to light of the water surface. A similar reduction in efficacy was also recorded in the case of other active ingredients, such as S-methoprene and *Bti*, which could be attributed to similar reasons with the dissipation of spinosad. Thus, spinosad, which was proved to be perhaps the most effective insecticide in the laboratory, was among the least effective insecticides in the semi-field tests, which underlines the need to combine laboratory bioassays with field evaluation protocols.

PDMS (Aquatain) was probably the most effective insecticide among the ones tested here, as it remained unaffected for a long period of time. The use of PDMS has been proposed in large areas, such as catchments, but also in the urban and suburban environment, where the use of other insecticides is not always possible. In a recent study, Rumbos and Athanassiou (2020) indicated that this formulation was effective for the control of the biotype tested here, but also for *Cx. pipiens* biotype *molestus*, even at concentrations that were lower than the label rate. In that study, the authors found that PDMS had a remarkable “speed of kill,” as all larvae were dead after 3 days of exposure in semi-field tests, while larval mortality ranged between 60 and 88% even from the first day (Rumbos and Athanassiou 2020). Previous laboratory and field/semi-field tests confirmed the high efficacy of this insecticide for additional species of the genera *Culex*, *Aedes*, and *Anopheles* (Cilek et al. 2020, Webb and Russell 2009, 2012). However, recent results indicate that there are certain factors that limit PDMS efficacy, such as rain (Drago et al. 2017, 2020), limitations that may exist in the case of other larvicides as well. At the same time, the combined application of PDMS with other active ingredients showed no additive effects, probably due to the increased PDMS efficacy (Rumbos and Athanassiou 2020). PDMS has been also found to be effective when applied at concentrations that are lower than its label rate and has an oviposition deterrence action (Nikolaidou et al. 2021).

Diflubenzuron and temephos showed similar insecticidal and residual levels for all series of bioassays. In general, we recorded that diflubenzuron was more “slow acting” in comparison with temephos, which can be attributed to its mode of action, given that IGRs are non-neurotoxic and require a certain period to act. A similar trend was also recorded in the case of S-methoprene, where, during the laboratory bioassays, there was an increase in mortality on the second week post-application. Nevertheless, for both diflubenzuron and temephos, the results of the present work show that they can provide a satisfactory level of control for about 3 weeks, which should be taken into account when these insecticides are used in “real-world” applications. Both diflubenzuron and temephos have been proved to be effective even at concentrations that were lower than the label rate, but their residual effect is drastically reduced (Michaelakis et al. 2018, 2020; Rumbos and Athanassiou 2020). Interestingly, temephos cannot be detected by mated females, and thus, this insecticide can be combined with oviposition pheromone, to attract more individuals, that will eventually die after exposure to this insecticide (Michaelakis et al. 2018). Our data show that the efficacy of temephos is comparable with that of diflubenzuron, one of the major mosquito larvicides that are currently in use in EU, and can still play a role in cases with high larval densities and control strategies that are based on the rotation of active ingredients to mitigate resistance development.

Considering the post-exposure measurements of the semi-field tests, especially those on which the insects were “incubated” for an additional period, we saw some interesting results on the surviving adults. Hence, for some of the combinations tested, we saw that there were some variations between sexes in adult survival. In fact, for some combinations, there were significant differences only in adult male survival. Still, we observed no adult deformities in the surviving adults, and no further effects in progeny production capacity of the adults that had emerged from surviving larvae. Nevertheless, switching the surviving individuals from exposure to field conditions to controlled laboratory conditions (incl. blood means) might have affected the eventual outcome in adult emergence and functionality. Specific interactions with the environment, as well as the larval instar that was used in the current study, may be responsible for these variations (Rumbos and Athanassiou 2020). There are previous studies that underline certain delayed effects on larvae of *Cx. pipiens* after short exposures to insecticides. For instance, recently, Nikolaidou et al. (2021) found that larvae of this species can eventually die even if they are exposed to PDMS for periods that are shorter than 1 h and then placed in untreated water. Additional experimental work is required to underline possible effects on fecundity and longevity of adult *Cx. pipiens* in relation with previous immature exposure to larvicides, especially in the case of insect growth regulators. Tran et al. (2018) noted that specific stressors, such as warming of the insecticide, can drastically differentiate progeny production capacity of the exposed adults of *Cx. pipiens*. This can be also the case in the current work, as the stressors were expressed much more vigorously in the semi-field tests, as compared with the laboratory bioassays.

Interestingly, the larvicides showed some deviations in terms of their efficacy among the 3 months examined, while, in general, efficacy was higher on May, in comparison with June and July. As noted above, elevated light exposures and higher temperatures may be responsible for these variations, which, in some of the cases tested, shortened the residual effect of temephos and diflubenzuron by 1 week. Indeed, the temperature levels prevailing were generally higher on June and July, as compared with May, which may partially explain these variations, given that temperature also affects the efficacy of certain mosquito larvicides and indirectly alters the interaction of the exposed individuals with the treated substrate (Kilpatrick et al. 2008; Ciota et al. 2014; Hariprasad and Shetty 2017; Tran et al. 2018). Still, our data demonstrate the wide range of conditions for which some larvicides retain their efficacy, despite deviations. Nevertheless, the results presented here correspond to the specific conditions and experimental setup, so generalizations should be avoided, although some key conclusions can be drawn.

In summary, the results of our work show that there are insecticides that are more effective than others for the control of *Cx. pipiens*, but the rank of the insecticides used in terms of their larvicidal efficacy can change according to the experimental protocol followed. Moreover, our semi-field data demonstrate the challenges that are to be taken into account when designing a “real-world” application scenario for the control of *Cx. pipiens* and the importance to carry out bioassays locally, in the area of interest. We also show that there are insecticides that have a good residual effect, such as temephos, diflubenzuron, and, especially, PDMS, and, thus, these active ingredients can be considered further in area-wide management strategies, where the repeated applications are not always possible.

## Data Availability

The authors confirm that the data supporting the findings of this study are available within the article.
